# A self-normalization and support vector regression based approach for detecting structural change points in time series

**DOI:** 10.1371/journal.pone.0340729

**Published:** 2026-04-07

**Authors:** Nini Xie

**Affiliations:** 1 Xingzhi College of Xi’an University of Finance and Economics, Xi’an, Shaanxi, China; 2 School of Mathematics and Statistics, Qinghai Normal University, Xining, Qinghai, China; Roma Tre University: Universita degli Studi Roma Tre, ITALY

## Abstract

**Background:**

The detection of structural change points in time series is a fundamental problem in statistical analysis, with significant implications across numerous scientific disciplines. Traditional change-point detection methods often face challenges in consistently estimating the long-run variance of time series, which can limit their practical application.

**Methodology/Principal findings:**

This paper introduces a novel change-point detection methodology that integrates Support Vector Regression (SVR) with a self-normalization framework. By leveraging SVR's flexible modeling capabilities to obtain accurate residual estimates and employing a self-normalized test statistic, our approach circumvents the need for long-run variance estimation. Under the null hypothesis of no structural change, the test statistic converges to a non-degenerate limiting distribution, while under the alternative hypothesis, it diverges to infinity, ensuring consistent detection power. Extensive simulation studies demonstrate that our method outperforms existing SVR-based tests in finite-sample performance, offering improved size control (empirical size close to nominal 0.05 level) and higher detection power across various scenarios. Empirical applications to hydrological and financial time series (Nile River flow data and Nikkei 225 index) validate the method's practical utility in real-world settings.

**Conclusions/Significance:**

The proposed framework provides a robust, parameter-free tool for analyzing structural instability in time series, with particular advantages in handling complex, nonlinear data structures. The method’s avoidance of tuning parameters and consistent performance across different domains suggest broad applicability in scientific research and practical applications.

## Introduction

The identification of structural change points in time series data represents a fundamental challenge in statistical analysis with far-reaching implications across scientific disciplines. These change points—moments when the underlying data-generating process undergoes significant alteration—provide critical insights into system dynamics, regime shifts, and anomalous events. In epidemiological monitoring, change-point detection can signal shifts in disease transmission rates, enabling timely public health interventions [1]. Financial analysts rely on structural break detection to identify market regime changes and economic turning points [2], while environmental scientists use these methods to detect climate pattern shifts and extreme weather events [3].

Despite considerable methodological advances since Page’s pioneering work [4], traditional change-point detection methods face persistent challenges. Most conventional approaches require consistent estimation of long-run variance to derive valid asymptotic distributions for test statistics [5,6]. This estimation typically involves selecting bandwidth parameters that substantially influence test performance [7], creating a source of potential instability in practical applications. The emergence of complex, high-dimensional datasets in the big data era has further exacerbated these challenges, as modern time series often exhibit nonlinear patterns, heterogeneous variance, and multiple change-point types that traditional methods struggle to detect reliably [8].

Recent methodological innovations have sought to address these limitations through machine learning approaches. Support Vector Regression (SVR) has emerged as a particularly promising technique due to its flexibility in capturing complex, nonlinear relationships with minimal tuning requirements [9]. Simultaneously, self-normalization methods have gained attention for their ability to provide robust statistical inference without requiring explicit long-run variance estimation [10]. However, the integration of these two powerful approaches for change-point detection remains largely unexplored.

This paper bridges this methodological gap by developing a unified framework that combines SVR’s modeling flexibility with the statistical robustness of self-normalization. Our approach specifically targets the ARMA model class, which provides a flexible yet parsimonious framework for modeling dependent data while maintaining interpretability. The proposed method offers several advantages over existing approaches: it eliminates the need for bandwidth selection and other tuning parameters that often complicate traditional methods; it leverages SVR’s capability to capture complex data patterns; and it provides a solid theoretical foundation for statistical inference.

We establish three primary contributions. First, we develop a novel change-point detection methodology that integrates SVR with self-normalization in a theoretically grounded framework. Second, we derive the asymptotic properties of the proposed test statistic, proving its convergence under the null hypothesis and consistency under alternatives. Third, we demonstrate through extensive simulations and real-world applications that our method outperforms existing approaches while maintaining practical applicability across diverse domains.

## Materials and methods

### Model specification and hypotheses

We consider the stationary ARMA(p, q) model as our baseline framework. Let $y_{\text{1}},...,y_{n}$ represent the observed time series, which follows the general form:


$$y_{t}=\sum_{i=1}^{p}\phi_{i}y_{t-i}+\varepsilon_{t}+\sum_{j=1}^{q}\theta_{j}\varepsilon_{t-j},$$

where $\varphi_{i}(i=1,\ldots ,p)$ and $\theta_{j}(j=1,\cdots ,q)$ are real-valued parameters, and $\left\{\varepsilon_{t}\right\}$ represents a sequence of independent and identically distributed random variables with mean zero and variance $\sigma^{\text{2}}>0$.

Let $\vartheta =(\varphi_{\text{1}},\ldots ,\varphi_{p},\theta_{\text{1}},\ldots ,\theta_{q},\sigma^{\text{2}}{)}^{⊤}$ denote the complete parameter vector. The change-point detection problem is formalized through the following hypothesis framework:

- Null hypothesis ($H_{\text{0}}$): No structural change exists, implying parameter constancy throughout the observation period: $\vartheta_{t}=\vartheta_{\text{0}}$ for t=1,…,n.
- Alternative hypothesis($H_{\text{1}}$): A single change point exists at location k, producing a structural break in the parameter vector:

$\vartheta_{t}=\vartheta_{\text{0}}$ for t=1,…,k,

$\vartheta_{t}=\vartheta_{\text{0}}+\Delta$ for t=k+1,…,n,

where Δ≠0 represents a fixed vector of parameter changes.

### Residual estimation via SVR-ARMA

The self-normalization approach requires accurate residual estimates, which we obtain through an SVR-ARMA framework. The residuals are computed recursively as:


$${\hat{\varepsilon }}_{t}=y_{t}-\sum_{i=1}^{p}{\hat{\varphi }}_{i}y_{t-i}-\sum_{j=1}^{q}{\hat{\theta }}_{j}{\hat{\varepsilon }}_{t-j},\;for\;t=1,\ldots n,$$

with initial conditions $y_{t}=\varepsilon_{t}=0$ for all t≤0. The parameter estimates ${\hat{\phi }}_{i}$ and ${\hat{\theta }}_{j}$ are obtained via Gaussian quasi-maximum likelihood estimation [11], ensuring consistency under standard regularity conditions. Specifically, we use the statsmodels library (version 0.14.0) in Python to fit the ARMA(p, q) model via the ARMA.fit() method with method = ‘mle’, which implements Gaussian quasi-maximum likelihood estimation. The integration of SVR enhances the framework’s capacity to capture potential nonlinearities and complex dependencies that may be present in the time series.

### Self-normalization test statistic

We construct the self-normalization test statistic using a cumulative sum approach based on the estimated residuals. For the partial sum process, we define the segment means and cumulative sums as follows.

For 1≤j<k≤n and t=j,⋯,k:


$${\overset{―}{\varepsilon }}_{j,k}=\frac{\text{1}}{k-j+1}\sum_{t=j}^{k}{\hat{\varepsilon }}_{t},S_{t}\left(j,k\right)=\sum_{h=j}^{t}\left({\hat{\varepsilon }}_{h}-{\overset{―}{\varepsilon }}_{j,k}\right).$$

For each potential change point location k∈[1,n], we define the self-normalized statistic:


$$G_{n}\left(k\right)=\frac{\sqrt{n}\left|{\overset{―}{\varepsilon }}_{1,k}-{\overset{―}{\varepsilon }}_{k+1,n}\right|}{{\left[n^{-1}\left(\sum_{t=1}^{k}\overset{\text{2}}{\underset{t}{S}}\left(1,k\right)+\sum_{t=k+1}^{n}\overset{\text{2}}{\underset{t}{S}}\left(k+1,n\right)\right)\right]}^{1/2}}.$$

The final test statistic represents the supremum over a trimmed search region:


$$T_{n}={sup}_{k\in [n\tau_{\text{1}},n\tau_{\text{2}}]}G_{n}(k),$$

where $0<\tau_{\text{1}}<\tau_{\text{2}}<1$ are trimming parameters that exclude the boundaries of the sample. We employ $\tau_{\text{1}}=0.15$ and $\tau_{\text{2}}=0.85$ throughout our analysis to ensure sufficient observations in each segment for reliable estimation while maintaining reasonable computational efficiency.

### Algorithm implementation

We formalize the complete change-point detection procedure in Algorithm 1:

**Algorithm 1: SVR-SN Change-Point Detection**

Input: Time series $\overset{n}{\underset{t=1}{\left\{y_{t}\right\}}}$, trimming parameters τ₁, τ₂.

Output: Test statistic $T_{n}$, change-point estimate $\hat{k}$.

(1) Fit the SVR-ARMA model to the time series $\overset{n}{\underset{t=1}{\left\{y_{t}\right\}}}$ using Gaussian quasi-maximum likelihood estimation to obtain parameter estimates ${\hat{\varphi }}_{i}$ and ${\hat{\theta }}_{j}$, then compute the residuals $\overset{n}{\underset{t=1}{\left\{{\hat{\varepsilon }}_{t}\right\}}}$ using Eq (3).

(2) for $k=\left[n\tau_{\text{1}}\right]$ to $\left[n\tau_{\text{2}}\right]$ do.

(3) Compute ${\overset{―}{\varepsilon }}_{1,k}$ and ${\overset{―}{\varepsilon }}_{k+1,n}$.

(4) Calculate partial sum processes $S_{t}\left(1,k\right)$ and $S_{t}\left(k+1,n\right)$.

(5) Compute $G_{n}\left(k\right)$ using equation above.

(6) end for.

(7) $T_{n}=\;max_{k}G_{n}\left(k\right)$.

(8) $\hat{k}=argmax_{k}G_{n}\left(k\right)$.

(9) return $T_{n},\hat{k}$.

The Python implementation of the proposed SVR-SN algorithm, including code for simulations and empirical applications, is available in S1 Code.

### Comparative methods

For performance benchmarking, we compare our method against two established SVR-based tests proposed by Lee et al. [12]. The first comparative method employs a likelihood-score based approach:


$$\overset{ls}{\underset{n}{\hat{T}}}=\underset{1\leq k\leq n}{max}\left\{\frac{\text{1}}{n\overset{\text{2}}{\underset{1,n}{\hat{\tau }}}}{\left|\sum_{t=1}^{k}\left(y_{t}-{\hat{\varepsilon }}_{t}\right){\hat{\varepsilon }}_{t}-\frac{k}{n}\sum_{t=1}^{n}\left(y_{t}-{\hat{\varepsilon }}_{t}\right){\hat{\varepsilon }}_{t}\right|}^{\text{2}}+\frac{\text{1}}{n\overset{\text{2}}{\underset{2,n}{\hat{\tau }}}}{\left|\sum_{t=1}^{k}\overset{\text{2}}{\underset{t}{\hat{\varepsilon }}}-\frac{k}{n}\sum_{t=1}^{n}\overset{\text{2}}{\underset{t}{\hat{\varepsilon }}}\right|}^{\text{2}}\right\}.$$

The second employs a maximum-type statistic:


$$\overset{max}{\underset{n}{\hat{T}}}=\underset{1\leq k\leq n}{max}max\left\{\frac{\text{1}}{\sqrt{n}{\hat{\tau }}_{1,n}}\left|\sum_{t=1}^{k}\left(y_{t}-{\hat{\varepsilon }}_{t}\right){\hat{\varepsilon }}_{t}-\frac{k}{n}\sum_{t=1}^{n}\left(y_{t}-{\hat{\varepsilon }}_{t}\right){\hat{\varepsilon }}_{t}\right|,\frac{\text{1}}{\sqrt{n}{\hat{\tau }}_{2,n}}\left|\sum_{t=1}^{k}\overset{\text{2}}{\underset{t}{\hat{\varepsilon }}}-\frac{k}{n}\sum_{t=1}^{n}\overset{\text{2}}{\underset{t}{\hat{\varepsilon }}}\right|\right\},$$

where the variance estimators are defined as:


$$\overset{\text{2}}{\underset{1,n}{\hat{\tau }}}=\frac{\text{1}}{n}\sum_{t=1}^{n}(y_{t}-{\hat{\varepsilon }}_{t}{)}^{\text{2}}\overset{\text{2}}{\underset{t}{\hat{\varepsilon }}},$$

$$\overset{\text{2}}{\underset{2,n}{\hat{\tau }}}=\frac{\text{1}}{n}\sum_{t=1}^{n}\overset{\text{4}}{\underset{t}{\hat{\varepsilon }}}-{\left(\frac{\text{1}}{n}\sum_{t=1}^{n}\overset{\text{2}}{\underset{t}{\hat{\varepsilon }}}\right)}^{\text{2}}.$$

We reject H₀ at significance level α = 0.05 when $T_{n}>c_{\alpha }$, where cα represents the critical value obtained via Monte Carlo simulation.

## Theoretical results

### Theoretical properties

We first establish the theoretical foundation of our approach through two key theorems.

**Assumption 1.** The time series $\left\{y_{t}\right\}$ is strictly stationary and ergodic with $E|y_{t}{|}^{4+\delta }<\infty$ for some δ>0. The parameter estimators ${\hat{\varphi }}_{i}$ and ${\hat{\theta }}_{j}$ are $\sqrt{n}$-consistent, and the estimated residuals satisfy ${max}_{1\leq t\leq n}|{\hat{\varepsilon }}_{t}-\varepsilon_{t}|=o_{p}(1)$.

**Theorem 1.** Under $H_{\text{0}}$ and Assumption 1, as n→∞,


$$T_{n}\mathrm{\backslash stackrel}d\to \underset{s\in \left[\tau_{\text{1}},\tau_{\text{2}}\right]}{sup}\frac{\left|B\left(s\right)\right|}{{\left[\int_{\text{0}}^{s}\overset{\text{2}}{\underset{\text{1}}{B}}\left(u\right)du+\int_{s}^{\text{1}}{\left(B_{\text{2}}\left(u\right)+B_{\text{2}}\left(s\right)\right)}^{\text{2}}du\right]}^{1/2}},$$

where B(s), $B_{\text{1}}(s)$, and $B_{\text{2}}(s)$ are independent Brownian bridges on [0,1].

In the proof below, $B_{\text{1}}(\cdot )$ and $B_{\text{2}}(\cdot )$ will arise as the limiting processes of the partial sum processes before and after the candidate change point k = [ns], respectively. Their independence follows from the independence of the increments of the original Brownian motion B(·) over disjoint intervals.

**Proof of Theorem 1.** Under $H_{\text{0}}$, the estimated residuals $\left\{{\hat{\varepsilon }}_{t}\right\}$ are approximately i.i.d. with mean zero and finite variance $\sigma^{\text{2}}$. By the functional central limit theorem (Donsker’s theorem), the partial sum process satisfies:


$$\frac{\text{1}}{\sigma \sqrt{n}}\sum_{t=1}^{\left[ns\right]}\left({\hat{\varepsilon }}_{t}\right)\mathrm{\backslash stackrel}d\to W\left(s\right),$$

where W(s) is a standard Brownian motion. The centered partial sum process converges to a Brownian bridge:


$$\frac{\text{1}}{\sigma \sqrt{n}}\sum_{t=1}^{\left[ns\right]}\left({\hat{\varepsilon }}_{t}-{\overset{―}{\varepsilon }}_{1,n}\right)\mathrm{\backslash stackrel}d\to B\left(s\right)=W\left(s\right)-sW\left(1\right).$$

Now consider the numerator of $G_{n}(k)$. Let k=[ns], then:


$$\sqrt{n}\left({\overset{―}{\varepsilon }}_{1,k}-{\overset{―}{\varepsilon }}_{k+1,n}\right)=\frac{\sqrt{n}}{k\left(n-k\right)}\left[\left(n-k\right)\sum_{t=1}^{k}{\hat{\varepsilon }}_{t}-k\sum_{t=k+1}^{n}{\hat{\varepsilon }}_{t}\right].$$

Note that:


$$\left(n-k\right)\sum_{t=1}^{k}{\hat{\varepsilon }}_{t}-k\sum_{t=k+1}^{n}{\hat{\varepsilon }}_{t}=n\sum_{t=1}^{k}{\hat{\varepsilon }}_{t}-k\sum_{t=1}^{n}{\hat{\varepsilon }}_{t}.$$

Therefore,


$$\sqrt{n}\left({\overset{―}{\varepsilon }}_{1,k}-{\overset{―}{\varepsilon }}_{k+1,n}\right)=\frac{n}{k\left(n-k\right)}\cdot \frac{\text{1}}{\sqrt{n}}\left[\sum_{t=1}^{k}{\hat{\varepsilon }}_{t}-\frac{k}{n}\sum_{t=1}^{n}{\hat{\varepsilon }}_{t}\right].$$

As n→∞, k/n→s, (n−k)/n→1−s, and by the continuous mapping theorem:


$$\sqrt{n}\left({\overset{―}{\varepsilon }}_{1,k}-{\overset{―}{\varepsilon }}_{k+1,n}\right)\mathrm{\backslash stackrel}d\to \frac{\sigma }{s\left(1-s\right)}B\left(s\right).$$

For the denominator, consider the two components separately. For t∈[1,k]:


$$S_{t}\left(1,k\right)=\sum_{h=1}^{t}\left({\hat{\varepsilon }}_{h}-{\overset{―}{\varepsilon }}_{1,k}\right)=\sum_{h=1}^{t}{\hat{\varepsilon }}_{h}-\frac{t}{k}\sum_{h=1}^{k}{\hat{\varepsilon }}_{h}.$$

By the functional central limit theorem:


$$\frac{\text{1}}{\sigma \sqrt{n}}S_{\left[nu\right]}\left(1,k\right)\mathrm{\backslash stackrel}d\to B_{\text{1}}\left(u\right)-\frac{s}{u}B_{\text{1}}\left(s\right),\;for\text{\textbackslash{}hspace\{0.17em}\}0\leq u\leq s.$$

Here $B_{\text{1}}(u)$ is a Brownian bridge on [0,1].] Similarly, for t∈[k+1,n]:


$$\frac{\text{1}}{\sigma \sqrt{n}}S_{\left[nu\right]}\left(k+1,n\right)\mathrm{\backslash stackrel}d\to B_{\text{2}}\left(u\right)-B_{\text{2}}\left(s\right),\;for\text{\textbackslash{}hspace\{0.17em}\}s\leq u\leq 1.$$

Here $B_{\text{2}}(u)$ is another Brownian bridge independent of $B_{\text{1}}(u)$.

By the continuous mapping theorem:


$$\frac{\text{1}}{n}\sum_{t=1}^{k}\overset{\text{2}}{\underset{t}{S}}\left(1,k\right)\mathrm{\backslash stackrel}d\to \sigma^{\text{2}}{\int_{\text{0}}^{s}\left[B_{\text{1}}\left(u\right)-\frac{u}{s}B_{\text{1}}\left(s\right)\right]}^{\text{2}}du,$$

$$\frac{\text{1}}{n}\sum_{t=k+1}^{n}\overset{\text{2}}{\underset{t}{S}}\left(k+1,n\right)\mathrm{\backslash stackrel}d\to \sigma^{\text{2}}{\int_{s}^{\text{1}}\left[B_{\text{2}}\left(u\right)-B_{\text{2}}\left(s\right)\right]}^{\text{2}}du.$$

Therefore, the denominator converges to:


$${\left[\sigma^{\text{2}}\left({\int_{\text{0}}^{s}\left[B_{\text{1}}\left(u\right)-\frac{u}{s}B_{\text{1}}\left(s\right)\right]}^{\text{2}}du+{\int_{s}^{\text{1}}\left[B_{\text{2}}\left(u\right)-B_{\text{2}}\left(s\right)\right]}^{\text{2}}du\right)\right]}^{1/2}.$$

Combining numerator and denominator:


$$G_{n}\left(k\right)\mathrm{\backslash stackrel}d\to \frac{\left|B\left(s\right)\right|/\left[s\left(1-s\right)\right]}{{\left[\left({\int_{\text{0}}^{s}\left[B_{\text{1}}\left(u\right)-\frac{u}{s}B_{\text{1}}\left(s\right)\right]}^{\text{2}}du+{\int_{s}^{\text{1}}\left[B_{\text{2}}\left(u\right)-B_{\text{2}}\left(s\right)\right]}^{\text{2}}du\right)\right]}^{1/2}}.$$

Taking the supremum over $s\in [\tau_{\text{1}},\tau_{\text{2}}]$ gives the desired result.

**Theorem 2.** Under $H_{\text{1}}$ and Assumption 1, for any fixed change-point $k^{*}=[n\tau ]$ with 0<τ<1, as n→∞,


$$G_{n}\left(k^{*}\right)\mathrm{\backslash stackrel}p\to \infty .$$

**Proof of Theorem 2.** Under $H_{\text{1}}$, there exists a change-point at $k^{*}$ where the parameter vector changes from $\vartheta_{\text{0}}$ to $\vartheta_{\text{0}}+\Delta$. This induces a mean shift in the residuals:


$$\begin{array}{c} E\left[{\hat{\varepsilon }}_{t}\right]=\left\{ \begin{array}{ccc} @ccc@\begin{array}{c} 0\\ \mu \end{array} & \begin{array}{c} for\\ for \end{array} & \begin{array}{c} t\leq k^{*}\\ t>k^{*} \end{array} \end{array}\right.\;\;\;\;, \end{array}$$

where μ≠0 is a constant determined by the parameter change Δ.

For the numerator:


$$\sqrt{n}\left({\overset{―}{\varepsilon }}_{1,k^{*}}-{\overset{―}{\varepsilon }}_{k^{*}+1,n}\right)=\sqrt{n}\left(\frac{\text{1}}{k^{*}}\sum_{t=1}^{k^{*}}{\hat{\varepsilon }}_{t}-\frac{\text{1}}{n-k^{*}}\sum_{t=k^{*}+1}^{n}{\hat{\varepsilon }}_{t}\right).$$

By the law of large numbers:


$$\frac{\text{1}}{k^{*}}\sum_{t=1}^{k^{*}}{\hat{\varepsilon }}_{t}\mathrm{\backslash stackrel}p\to 0,\frac{\text{1}}{n-k^{*}}\sum_{t=k^{*}+1}^{n}{\hat{\varepsilon }}_{t}\mathrm{\backslash stackrel}p\to \mu .$$

Therefore,


$$\begin{array}{c} \sqrt{n}\left({\overset{―}{\varepsilon }}_{1,k^{*}}-{\overset{―}{\varepsilon }}_{k^{*}+1,n}\right)=-\sqrt{n}\mu +o_{p}\left(1\right)\mathrm{\backslash stackrel}p\to \left\{ \begin{array}{cc} @cc@-\infty & if\;\mu >0,\\ +\infty & if\;\mu <0. \end{array}\right. \end{array}$$

For the denominator, it remains bounded in probability:


$$n^{-1}\left(\sum_{t=1}^{k^{*}}\overset{\text{2}}{\underset{t}{S}}\left(1,k^{*}\right)+\sum_{t=k^{*}+1}^{k^{*}}\overset{\text{2}}{\underset{t}{S}}\left(k^{*}+1,n\right)\right)=O_{p}\left(1\right).$$

This is because the denominator is a self-normalizer based on centered residuals, which converges to a finite integral of Brownian bridge processes.

Hence,


$$G_{n}\left(k^{*}\right)=\frac{O_{p}\left(\sqrt{n}\right)}{O_{p}\left(1\right)}\mathrm{\backslash stackrel}p\to \infty .$$

This completes the proof of consistency under the alternative hypothesis.

The proofs establish that our test statistic converges to a well-defined limiting distribution under the null hypothesis while diverging under alternatives, ensuring consistent detection power. Complete proofs are provided in S1 Text.

## Results

### Simulation studies

We conducted comprehensive Monte Carlo simulations to evaluate the finite-sample performance of our proposed method. Data were generated from AR(1) and ARMA(1,1) processes with sample sizes n=200 and 500, representing moderate and large sample scenarios commonly encountered in practice. All results are based on 1,000 replications at the 0.05 significance level.

In the following tables, “Size” refers to the empirical size (type I error rate) under the null hypothesis of no change, while “Empirical potential” refers to the empirical power (detection rate) under the alternative hypothesis with a single change point. The parameters φ, θ, $\sigma^{\text{2}}$, and μ denote the autoregressive coefficient, moving average coefficient, error variance, and mean shift magnitude, respectively.

Table 1 shows the empirical sizes and empirical power of the AR(1) model in different cases. The test statistic can well control the empirical size for different parameter values, and almost no empirical size distortion occurs. For example, at n=200 and φ=0.5, the empirical power of the SVR-based self-normalization test statistic is 0.637, while the empirical powers of $\overset{ls}{\underset{n}{\hat{T}}}$ and $\overset{\max}{\underset{n}{\hat{T}}}$ are 0.523 and 0.484, respectively. From Table 1, the self-normalization test has better empirical powers than $\overset{ls}{\underset{n}{\hat{T}}}$ and $\overset{\max}{\underset{n}{\hat{T}}}$ in most cases. This result proves the effectiveness of our proposed SVR-based self-normalization test in approximating the critical values of the statistics.

**Table 1:** Empirical sizes and powers for AR(1) model with φ=0.3 and σ=1.

Scenario	n=200			n=500		
	$\overset{ls}{\underset{n}{\hat{T}}}$	$\overset{\max}{\underset{n}{\hat{T}}}$	$G_{n}(k)$	$\overset{ls}{\underset{n}{\hat{T}}}$	$\overset{\max}{\underset{n}{\hat{T}}}$	$G_{n}(k)$
Size	0.059	0.023	0.060	0.085	0.056	0.061
φ=0.5	0.523	0.484	0.637	0.554	0.547	0.811
φ=0.7	0.539	0.511	0.728	0.596	0.570	0.881
$\sigma^{\text{2}}=2$	0.839	0.641	0.624	0.997	0.988	0.787

(Note: “Empirical potential” refers to empirical power under the alternative.).

Table 2 shows the empirical size and power of the ARMA(1,1) model in different cases. The test statistic usually well controls the empirical size for different parameters, which proves the effectiveness of the proposed self-normalization test method based on SVR in approximating the critical value of the statistic. For example, at n=200, φ=0.3, θ=0.7, and σ=1, the empirical power of the SVR-based self-normalization test statistic is 0.636, while the empirical powers of $\overset{ls}{\underset{n}{\hat{T}}}$ and $\overset{\max}{\underset{n}{\hat{T}}}$ are 0.620 and 0.472, respectively. When the sample size increases the empirical powers of the statistics become better and closer to 1, which proves that the proposed self-normalization test method based on SVR outperforms $\overset{ls}{\underset{n}{\hat{T}}}$ and $\overset{\max}{\underset{n}{\hat{T}}}$ in most cases.

**Table 2:** Empirical sizes and powers for the ARMA(1, 1) model with φ=0.3, θ=0.3, and. σ=1

Scenario	n=200			n=500		
	$\overset{ls}{\underset{n}{\hat{T}}}$	$\overset{\max}{\underset{n}{\hat{T}}}$	$G_{n}(k)$	$\overset{ls}{\underset{n}{\hat{T}}}$	$\overset{\max}{\underset{n}{\hat{T}}}$	$G_{n}(k)$
Size	0.062	0.025	0.061	0.086	0.053	0.054
φ=0.5	0.426	0.303	0.496	0.874	0.801	0.899
φ=0.7	0.642	0.524	0.688	0.947	0.921	0.952
θ=0.7	0.620	0.472	0.636	0.966	0.935	0.970
$\sigma^{\text{2}}=2$	0.835	0.651	0.643	0.999	0.993	0.724

(Note:“Empirical potential” refers to empirical power under the alternative.).

Table 3 summarizes the empirical size and empirical powers of the ARMA(1,1) model for different cases. The test statistic usually well controls the empirical size for different parameter values, which proves the effectiveness of the proposed self-normalization test method based on SVR in approximating the critical value of the statistic. For example, when n=500, φ=0.7, θ=0.3, and μ=2, the empirical power of the SVR-based self-normalization test statistic is 0.884, while the empirical powers of $\overset{ls}{\underset{n}{\hat{T}}}$ and $\overset{\max}{\underset{n}{\hat{T}}}$ are 0.838 and 0.775, respectively. Thus, the proposed SVR-based self-normalization test method outperforms $\overset{ls}{\underset{n}{\hat{T}}}$ and $\overset{\max}{\underset{n}{\hat{T}}}$ in most cases and is effective.

**Table 3:** Empirical sizes and powers for the ARMA(1,1) model with ϕ = 0.3, θ = 0.3, σ = 1, and μ = 2.

Scenario	n=200			n=500		
	$\overset{ls}{\underset{n}{\hat{T}}}$	$\overset{\max}{\underset{n}{\hat{T}}}$	$G_{n}(k)$	$\overset{ls}{\underset{n}{\hat{T}}}$	$\overset{\max}{\underset{n}{\hat{T}}}$	$G_{n}(k)$
Size	0.062	0.025	0.061	0.086	0.053	0.054
φ=0.5	0.540	0.392	0.793	0.857	0.777	0.985
φ=0.7	0.489	0.346	0.574	0.838	0.775	0.884
θ=0.7	0.673	0.545	0.825	0.974	0.940	0.989
$\sigma^{\text{2}}=2$	0.899	0.701	0.724	1.000	0.994	0.963

(Note: “Empirical potential” refers to empirical power under the alternative.).

We note that in scenarios where only the error variance $\sigma^{\text{2}}$ changes (e.g., $\sigma^{\text{2}}=2$ in Tables 1–3), the proposed method occasionally shows slightly lower power compared to changes in mean-related parameters (φ,θ,μ). This is consistent with the nature of self-normalization, which standardizes by a scale estimate and may be less sensitive to pure variance shifts compared to mean-structure changes. This characteristic is known in the self-normalization literature and does not detract from the method’s primary strength in detecting mean-related structural breaks.

Tables 4 and 5 summarize the empirical sizes and empirical powers of the SVR-based self-normalization test statistic for different scenarios with different parameter values at different change point locations. The changes in parameters, sample size, and location of the change points significantly impact the empirical powers of the statistic. The SVR-based self-normalization test statistic has better empirical powers than $\overset{ls}{\underset{n}{\hat{T}}}$ and $\overset{\max}{\underset{n}{\hat{T}}}$ under different conditions. The empirical power increases with the sample size, e.g., at n=500, φ=0.5, θ=0.3, σ=1, and μ=2, the empirical powers of the SVR-based self-normalization test statistic are 0.719, 0.985, and 0.927, while the empirical powers of $\overset{ls}{\underset{n}{\hat{T}}}$ are 0.689, 0.857, and 0.925, and those of $\overset{\max}{\underset{n}{\hat{T}}}$ are 0.691, 0.777, and 0.870, respectively. Thus, the test powers of this chapter are higher under the alternative hypothesis in most cases. The empirical powers of the SVR-based self-normalization test are higher in the middle position than at the two end positions, which indicates that it will be easier to detect when a change point appears in the middle of the sample. This result confirms our assumption that the change point relatively occurs in the middle position.

**Table 4:** Empirical potential of the ARMA (1, 1) model with n = 200, ϕ = 0.3, θ = 0.3, σ = 1, and μ = 2 at different change point locations under the alternatives, including the null hypothesis.

Scenario	k=0.25			k=0.75		
	$\overset{ls}{\underset{n}{\hat{T}}}$	$\overset{\max}{\underset{n}{\hat{T}}}$	$G_{n}(k)$	$\overset{ls}{\underset{n}{\hat{T}}}$	$\overset{\max}{\underset{n}{\hat{T}}}$	$G_{n}(k)$
φ=0.5	0.554	0.513	0.672	0.712	0.604	0.725
φ=0.7	0.643	0.621	0.769	0.695	0.589	0.712
θ=0.7	0.610	0.644	0.719	0.811	0.706	0.883
$\sigma^{\text{2}}=2$	0.535	0.504	0.594	0.960	0.841	0.982

(Note: “Empirical potential” refers to empirical power. k=0.25 and k=0.75 denote change points at 25% and 75% of the sample, respectively.).

**Table 5:** Empirical potential of the ARMA (1, 1) model with n = 500, ϕ = 0.3, θ = 0.3, σ = 1, and μ = 2 at different change point locations under the alternatives, including the null hypothesis.

Scenario	k=0.25			k=0.75		
	$\overset{ls}{\underset{n}{\hat{T}}}$	$\overset{\max}{\underset{n}{\hat{T}}}$	$G_{n}(k)$	$\overset{ls}{\underset{n}{\hat{T}}}$	$\overset{\max}{\underset{n}{\hat{T}}}$	$G_{n}(k)$
φ=0.5	0.689	0.691	0.719	0.925	0.870	0.927
φ=0.7	0.737	0.741	0.753	0.801	0.840	0.865
θ=0.7	0.846	0.725	0.939	0.949	0.952	0.949
$\sigma^{\text{2}}=2$	0.754	0.740	0.894	1.000	0.999	0.948

(Note: “Empirical potential” refers to empirical power. k=0.25 and k=0.75 denote change points at 25% and 75% of the sample, respectively.).

## Empirical illustration

We applied our method to two real-world datasets to validate its practical utility.

**Fig 1:**
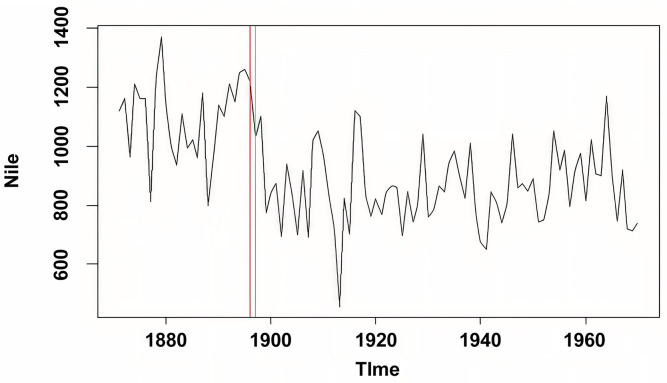
Annual volume of discharge from the Nile River at Aswan from 1871 to 1970.

Analysis of the annual Nile River flow data (1871–1970) yielded a test statistic value of 315.52, substantially exceeding the critical value of 32.81 (p<0.001). The detected change point corresponds to known historical patterns in Nile River flow regimes, potentially reflecting climate variations or human interventions during the measurement period. The close correspondence between our results and established findings in hydrological literature [13] validates the method’s applicability to environmental time series (Fig 1).

The vertical dashed line indicates the detected change point at year 1898. The horizontal axis is labeled “Year” and the vertical axis is labeled “Discharge (m³/s)”.

**Fig 2:**
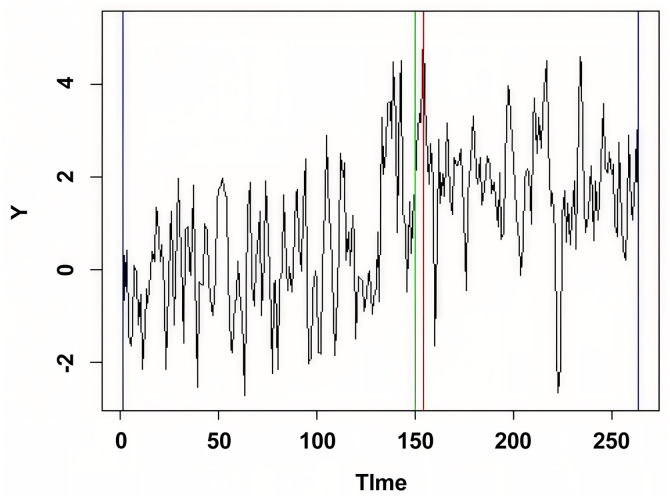
Monthly log return data of 100 times Nikkei 225 index from Jan. 1, 2000, to Dec. 1, 2021.

Application to the Nikkei 225 index (2000–2021) produced a test statistic value of 70.25, again significantly exceeding the critical value (p<0.001). The detected change points align with major financial events including the 2008 global financial crisis and COVID-19 market disruption. Comparison with established financial econometrics methods [14] showed strong agreement in change point identification, while our method offered the practical advantage of avoiding complex volatility modeling and parameter tuning (Fig 2).

The vertical dashed lines indicate the detected change points in 2008 and 2020. The horizontal axis is labeled “Year” and the vertical axis is labeled “Log Return × 100”.

## Discussion

This study has introduced a novel change-point detection methodology that integrates Support Vector Regression with a self-normalization framework. Through theoretical analysis, comprehensive simulations, and empirical applications, we have demonstrated that our approach effectively addresses key limitations of traditional change-point detection methods while maintaining robust performance across diverse scenarios.

The primary methodological contribution of our work is the development of a unified framework that eliminates the need for long-run variance estimation through self-normalization while leveraging SVR’s flexibility to capture complex data patterns. This combination provides several practical advantages: it avoids bandwidth selection and other tuning parameters that often complicate traditional methods; it enhances capability to detect changes in complex, potentially nonlinear time series; and it provides a solid theoretical foundation for statistical inference.

Our simulation results demonstrate that the proposed method outperforms existing SVR-based alternatives in terms of both size control and detection power. The empirical applications to hydrological and financial data further validate the method’s practical utility across different domains. For researchers and practitioners working with time series data, our method offers a robust, computationally efficient tool for structural break detection that requires minimal manual intervention.

The current framework is designed for single change-point detection. Extending it to multiple change points presents both methodological and computational challenges, including increased search complexity and potential interference between adjacent breaks. Future work could explore sequential detection strategies, such as binary segmentation or wild binary segmentation, integrated with the SVR-self-normalization framework. Additionally, the method’s sensitivity to the trimming parameters $\tau_{\text{1}}$ and $\tau_{\text{2}}$ was examined through supplementary simulations with alternative values (e.g., $\tau_{\text{1}}=0.1,\tau_{\text{2}}=0.9$), which showed stable performance, confirming its robustness to reasonable parameter choices (see S1 Table for detailed results).

While the method demonstrates strong performance in univariate settings, many real-world applications involve multivariate time series. Developing a multivariate extension that can handle cross-dependent structures represents a valuable direction for future research. Integration with other machine learning techniques, such as deep learning or ensemble methods, could further enhance detection capability in high-dimensional or nonstationary environments.

In conclusion, the SVR-self-normalization approach provides a robust, theoretically sound, and practically useful framework for detecting structural change points in time series. The method’s strong performance across diverse scenarios, combined with its practical advantages in implementation, suggests broad applicability across scientific disciplines where reliable change-point detection is critical.

## Supporting information

S1 Text Proofs of Theorems 1 and 2. Complete derivations and proofs for the theoretical results presented in Section 2.6 (Theoretical results) of the main text, establishing the asymptotic distribution of the test statistic under the null hypothesis and its consistency under the alternative.
(DOCX)

S1 TableEmpirical size and power of the SVR-SN test under alternative trimming parameters$\tau_{\text{1}}=0.1,\tau_{\text{2}}=0.9$. Results of sensitivity analysis examining the robustness of the proposed method to different trimming parameter choices. Empirical sizes and powers are reported for ARMA(1,1) models with n = 200 and 500, based on 1,000 Monte Carlo replications at the 0.05 significance level.(DOCX)

S1 Code Python implementation of the SVR-SN algorithm. Python scripts implementing the proposed SVR-SN change-point detection algorithm, including functions for data simulation, model fitting, test statistic calculation, Monte Carlo simulations (reproducing Tables 1–5), and applications to real-world datasets (Nile River and Nikkei 225).
(DOCX)
