# Supplementary material for: A self-normalization and support vector regression based approach for detecting structural change points in time series
Source: PLoS One. 2026 Apr 7;21(4):e0340729. doi: 10.1371/journal.pone.0340729 (PMC13056206; doi:10.1371/journal.pone.0340729)
Supplement: S1 Text — Complete derivations and proofs for the theoretical results presented in Section 2.6 (Theoretical results) of the main text, establishing the asymptotic distribution of the test statistic under the null hypothesis and its consistency under the alternative. (DOCX) [file pone.0340729.s001.docx]

**Proof S1. Complete proofs of Theorems 1 and 2.**

This document provides the detailed derivations and proofs for the theoretical results presented in Section 2.6 (Theoretical results) of the main text.

**Proof of Theorem 1.**

Under $H_{0}$ and Assumption 1, we need to show that as $n\to\infty$,

$$T_{n}=\sup_{k\in[n\tau_{1},n\tau_{2}]}G_{n}(k)\overset{d}{\to}\sup_{s\in[\tau_{1},\tau_{2}]}\frac{\mid B(s)-sB(1)\mid}{\sqrt{s(1-s)\left[ \int_{0}^{1} (B_{1}(u))^{2}du+\int_{0}^{1} (B_{2}(u))^{2}du \right]}},$$

Where $B(\cdot)$, $B_{1}(\cdot)$, and $B_{2}(\cdot)$ are independent Brownian bridges on $\left[ 0 , 1 \right]$.

*Step 1: Convergence of the numerator.*

Under the null hypothesis, the estimated residuals $\left\{ \hat{\varepsilon}_{t} \right\}$ are approximately i.i.d. with mean zero and finite variance $\sigma^{2}$. Define the partial sum process $S_{k}=\sum_{t=1}^{k} \hat{\varepsilon}_{t}$. By the Functional Central Limit Theorem (Donsker's Theorem),

$$\frac{1}{\sqrt{n}\sigma}S_{\left\lfloor ns \right\rfloor}\overset{d}{\to}W(s),$$

Where $W(s)$ is a standard Brownian motion. The centered partial sum process converges to a Brownian bridge:

$$\frac{1}{\sqrt{n}\sigma}\left( S_{\left\lfloor ns \right\rfloor} - \frac{\left\lfloor ns \right\rfloor}{n}S_{n} \right)\overset{d}{\to}B(s)-sB(1)\equiv B^{*}(s),$$

Where $B(s)=W(s)-sW(1)$ is a Brownian bridge. Let $k=\lfloor ns\rfloor$. The numerator of $G_{n}(k)$ is:

$$\text{Num}_{n}(k)=\mid\sum_{t=1}^{k} \hat{\varepsilon}_{t}-\frac{k}{n}\sum_{t=1}^{n} \hat{\varepsilon}_{t}\mid=\mid S_{k}-\frac{k}{n}S_{n}\mid.$$

Therefore,

$$\frac{1}{\sqrt{n}\sigma}\text{Num}_{n}(\lfloor ns\rfloor)\overset{d}{\to}\mid B^{*}(s)\mid.$$

*Step 2: Convergence of the denominator.*

The denominator is a self-normalizer. We analyze the two components separately. For the first segment ($t=1,\ldots,k$):

$$V_{1,n}^{2}(k)=\sum_{t=1}^{k} \left( \hat{\varepsilon}_{t} - \frac{1}{k}S_{k} \right)^{2}.$$

Define the process within the first segment: for $u\in[0,1]$,

$$\frac{1}{\sqrt{k}\sigma}\left( S_{\left\lfloor ku \right\rfloor} - \frac{\left\lfloor ku \right\rfloor}{k}S_{k} \right)\overset{d}{\to}B_{1}(u),$$

Where $B_{1}(u)$ is a Brownian bridge obtained from the increments of $W(s)$ on $\left[ 0 , s \right]$. By the continuous mapping theorem,

$$\frac{1}{k\sigma^{2}}V_{1,n}^{2}(\lfloor ns\rfloor)=\frac{1}{k}\sum_{t=1}^{k} \left( \frac{1}{\sigma}\left( \hat{\varepsilon}_{t} - \frac{1}{k}S_{k} \right) \right)^{2}\overset{d}{\to}\int_{0}^{1} (B_{1}(u))^{2}du.$$

Similarly, for the second segment ($t=k+1,\ldots,n$):

$$V_{2,n}^{2}(k)=\sum_{t=k+1}^{n} \left( \hat{\varepsilon}_{t}-\frac{1}{n-k}(S_{n}-S_{k}) \right)^{2}.$$

Define the process within the second segment: for $u\in[0,1]$,

$$\frac{1}{\sqrt{n-k}\sigma}\left( S_{k+\lfloor(n-k)u\rfloor}-S_{k}-\frac{\left\lfloor(n-k)u \right\rfloor}{n-k}(S_{n}-S_{k}) \right)\overset{d}{\to}B_{2}(u),$$

Where $B_{2}(u)$ is a Brownian bridge independent of $B_{1}(u)$, arising from the increments of $W(s)$ on $\left[ s , 1 \right]$. Consequently,

$$\frac{1}{(n-k)\sigma^{2}}V_{2,n}^{2}(\lfloor ns\rfloor)\overset{d}{\to}\int_{0}^{1} (B_{2}(u))^{2}du.$$

*Step 3: Joint convergence and final ratio.*

The processes $B_{1}(\cdot)$ and $B_{2}(\cdot)$ are independent because they are built from increments of the original Brownian motion$W(\cdot)$over the disjoint intervals $\left[ 0 , s \right]$ and $\left[ s , 1 \right]$. Combining the results from Steps 1 and 2 via the continuous mapping theorem for the ratio, we have:

$$G_{n}(\lfloor ns\rfloor)=\frac{\text{Num}_{n}(\lfloor ns\rfloor)/\sqrt{n}}{\sqrt{\frac{1}{n}V_{1,n}^{2}(\lfloor ns\rfloor)+\frac{1}{n}V_{2,n}^{2}(\lfloor ns\rfloor)}}\overset{d}{\to}\frac{\mid B^{*}(s)\mid}{\sqrt{s\int_{0}^{1} (B_{1}(u))^{2}du+(1-s)\int_{0}^{1} (B_{2}(u))^{2}du}}.$$

Noting that $B^{*}(s)=\sqrt{s(1-s)}\tilde{B}(s)$ for some standard Brownian bridge $\tilde{B}(s)$ in distribution, and applying the continuous mapping theorem for the supremum over $s\in[\tau_{1},\tau_{2}]$, we obtain the stated result in the main text (which presents an equivalent form of the limit).

**Proof of Theorem 2.**

Under $H_{1}$, there exists a true change-point at $k^{*}=\lfloor n\tau\rfloor$ with $0<\tau<1$ such that the parameter vector shifts from $\vartheta_{0}$ to $\vartheta_{0}+\Delta$, $\Delta\neq0$. This induces a persistent shift in the mean of the (estimated) residuals. Specifically, there exists a constant $\mu\neq0$ (which depends on $\Delta$ and the model) such that for $t>k^{*}$, $E(\hat{\varepsilon}_{t})\approx\mu$.

*Step 1: Divergence of the numerator.*

Consider the numerator of $G_{n}(k^{*})$:

$$\text{Num}_{n}(k^{*})=\mid\sum_{t=1}^{k^{*}} \hat{\varepsilon}_{t}-\frac{k^{*}}{n}\sum_{t=1}^{n} \hat{\varepsilon}_{t}\mid=\mid\left( 1 - \frac{k^{*}}{n} \right)\sum_{t=1}^{k^{*}} \hat{\varepsilon}_{t}-\frac{k^{*}}{n}\sum_{t=k^{*}+1}^{n} \hat{\varepsilon}_{t}\mid.$$

Under the alternative, the second sum experiences a mean shift:

$$\frac{1}{n-k^{*}}\sum_{t=k^{*}+1}^{n} \hat{\varepsilon}_{t}\overset{P}{\to}\mu.$$

Therefore,

$$\frac{1}{n}\sum_{t=k^{*}+1}^{n} \hat{\varepsilon}_{t}\overset{P}{\to}(1-\tau)\mu.$$

Meanwhile, $\frac{1}{k^{*}}\sum_{t=1}^{k^{*}} \hat{\varepsilon}_{t}\overset{P}{\to}0$. Substituting these probability limits yields:

$$\frac{1}{n}\text{Num}_{n}(k^{*})\overset{P}{\to}\mid0-\tau(1-\tau)\mu\mid=\tau(1-\tau)\mid\mu\mid>0.$$

Hence, $\text{Num}_{n}(k^{*})=O_{p}(n)$, diverging at rate $n$.

*Step 2: Boundedness of the denominator.*
The denominator is a self-normalizer based on centered residuals. Regardless of the mean shift, the centered second moments remain stochastically bounded. More formally,

$$\frac{1}{n}V_{1,n}^{2}(k^{*})=\frac{1}{n}\sum_{t=1}^{k^{*}} \left( \hat{\varepsilon}_{t} - \frac{1}{k^{*}}\sum_{i=1}^{k^{*}} \hat{\varepsilon}_{i} \right)^{2}=O_{p}(1),$$

$$\frac{1}{n}V_{2,n}^{2}(k^{*})=\frac{1}{n}\sum_{t=k^{*}+1}^{n} \left( \hat{\varepsilon}_{t} - \frac{1}{n-k^{*}}\sum_{i=k^{*}+1}^{n} \hat{\varepsilon}_{i} \right)^{2}=O_{p}(1).$$

Therefore, the denominator $\sqrt{\frac{1}{n}V_{1,n}^{2}(k^{*})+\frac{1}{n}V_{2,n}^{2}(k^{*})}=O_{p}(1)$.

*Step 3: Consistency of the test.*
Combining the results,

$$G_{n}(k^{*})=\frac{O_{p}(n)}{O_{p}(1)}\to\infty\text{in probability, as }n\to\infty.$$

Since the test statistic $T_{n}\geq G_{n}(k^{*})$, it follows that $T_{n}\overset{P}{\to}\infty$ under $H_{1}$. This guarantees that for any fixed critical value $c_{\alpha}$, $P(T_{n}>c_{\alpha})\to1$ as $n\to\infty$, establishing the consistency of the test. ∎
