# Supplementary material for: A self-normalization and support vector regression based approach for detecting structural change points in time series
Source: PLoS One. 2026 Apr 7;21(4):e0340729. doi: 10.1371/journal.pone.0340729 (PMC13056206; doi:10.1371/journal.pone.0340729)
Supplement: S1 Table — τ1=0.1,τ2=0.9. Results of sensitivity analysis examining the robustness of the proposed method to different trimming parameter choices. Empirical sizes and powers are reported for ARMA(1,1) models with n = 200 and 500, based on 1,000 Monte Carlo replications at the 0.05 significance level. (DOCX) [file pone.0340729.s002.docx]

**S1 Table. Empirical size and power of the SVR-SN test under alternative trimming parameters** $\left( \boldsymbol{\tau}_{\mathbf{1}}\mathbf{=0.10,}\boldsymbol{\tau}_{\mathbf{2}}\mathbf{=0.90} \right)$**.**

Results are based on 1,000 Monte Carlo replications at the nominal 0.05 level. Data are generated from an ARMA (1, 1) model with baseline parameters $\phi=0.3$, $\theta=0.3$, $\sigma=1$. “Size” denotes the empirical size under $H_{0}$. “Power” scenarios involve a single change point at $\tau=0.5$ with the specified parameter shift.

| Scenario | n = 200 | n = 500 |
| --- | --- | --- |
| Size | 0.058 | 0.062 |
| $\phi=0.7$ | 0.701 | 0.953 |
| $\theta=0.7$ | 0.665 | 0.968 |
| $\sigma^{2}=2$ | 0.655 | 0.748 |
| $\mu=2$ | 0.815 | 0.991 |

Note: The performance of the test with trimming parameters $\left( \tau_{1}=0.10,\tau_{2}=0.90 \right)$ is very similar to the baseline setting $\left( \tau_{1}=0.15,\tau_{2}=0.85 \right)$ reported in the main text (Tables 2 & 3). The empirical size remains close to the nominal 0.05 level, and power increases with sample size. This indicates that the proposed method is robust to reasonable variations in the choice of trimming parameters.
