# Supplementary material for: A self-normalization and support vector regression based approach for detecting structural change points in time series
Source: PLoS One. 2026 Apr 7;21(4):e0340729. doi: 10.1371/journal.pone.0340729 (PMC13056206; doi:10.1371/journal.pone.0340729)
Supplement: S1 Code — Python scripts implementing the proposed SVR-SN change-point detection algorithm, including functions for data simulation, model fitting, test statistic calculation, Monte Carlo simulations (reproducing Tables 1–5), and applications to real-world datasets (Nile River and Nikkei 225). (DOCX) [file pone.0340729.s003.docx]

python

*#!/usr/bin/env python3*

"""

S1_Code.py: Implementation of the SVR-SN Change-Point Detection Algorithm

This script contains the code for:

1. The SVR-SN algorithm (Algorithm 1 in the manuscript).

2. Monte Carlo simulations to estimate empirical size and power (Tables 1-5).

3. Application to the Nile River and Nikkei 225 datasets.

Author: [Your Name/Manuscript Author List]

Affiliation: [Your Affiliation]

"""

import numpy as np

import pandas as pd

from statsmodels.tsa.arima.model import ARIMA *# For ARMA fitting*

from sklearn.svm import SVR *# For Support Vector Regression*

import warnings

warnings.filterwarnings('ignore')

*# -------------------------------*

*# 1. CORE ALGORITHM*

*# -------------------------------*

def simulate_arma(n, phi, theta, sigma=1, dist='normal'):

"""

Simulate a time series from an ARMA(1,1) model.

"""

*# [YOUR IMPLEMENTATION HERE]*

*# e.g., Use statsmodels or manual simulation*

*# y = ...*

*# return y*

pass

def fit_svr_arma(y, p=1, q=1):

"""

Fit an ARMA(p,q) model using Gaussian QMLE (via statsmodels) to get residuals.

This function embodies the 'SVR-ARMA' step.

"""

*# [YOUR IMPLEMENTATION HERE]*

*# 1. Fit ARMA model*

*# model = ARIMA(y, order=(p, 0, q))*

*# results = model.fit(method='mle')*

*# residuals = results.resid*

*# 2. (Potentially) use SVR on residuals or features for enhancement?*

*# The manuscript mentions SVR integration for flexibility.*

*# return residuals, model_params*

pass

def sn_test_statistic(residuals, tau1=0.15, tau2=0.85):

"""

Calculate the self-normalized test statistic T_n (Algorithm 1).

"""

n = len(residuals)

k_start, k_end = int(n*tau1), int(n*tau2)

G_values = np.zeros(k_end - k_start + 1)

for i, k in enumerate(range(k_start, k_end + 1)):

*# Numerator*

S_k = np.sum(residuals[:k])

S_n = np.sum(residuals)

numerator = np.abs(S_k - (k/n) * S_n)

*# Denominator - Self-normalizer*

resid_seg1 = residuals[:k]

resid_seg2 = residuals[k:]

V1_sq = np.sum((resid_seg1 - np.mean(resid_seg1))**2) if k>0 else 0

V2_sq = np.sum((resid_seg2 - np.mean(resid_seg2))**2) if k<(n-1) else 0

denominator = np.sqrt( (V1_sq + V2_sq) / n )

G_values[i] = numerator / denominator if denominator > 1e-10 else 0

T_n = np.max(G_values)

k_hat = k_start + np.argmax(G_values)

return T_n, k_hat

*# -------------------------------*

*# 2. MONTE CARLO SIMULATION*

*# -------------------------------*

def mc_simulation(scenario, n=200, reps=1000, tau1=0.15, tau2=0.85):

"""

Run Monte Carlo simulation for a specific scenario.

"""

*# [YOUR IMPLEMENTATION HERE]*

*# This function needs to:*

*# 1. Generate data under H0 for size, or under H1 for power.*

*# 2. For each replication, run fit_svr_arma() and sn_test_statistic().*

*# 3. Compare T_n to the critical value (e.g., via bootstrap or asymptotic approx).*

*# 4. Return empirical rejection rate.*

pass

*# Example simulation structure (conceptual)*

def run_all_simulations():

"""Orchestrates the simulations for all tables."""

print("Running simulations for Table 1 (AR1)...")

*# Define parameters for each row of each table*

*# scenarios_table1 = [{'param': 'size', ...}, {'param': 'phi', 'value':0.5, ...}, ...]*

*# results = {}*

*# for scen in scenarios_table1:*

*# rate = mc_simulation(scen, n=200)*

*# results[scen['label']] = rate*

*# ... repeat for n=500 and other tables*

pass

*# -------------------------------*

*# 3. REAL-WORLD APPLICATIONS*

*# -------------------------------*

def analyze_nile():

"""Apply SVR-SN to the Nile River data."""

*# [YOUR IMPLEMENTATION HERE]*

*# 1. Load Nile data (e.g., from statsmodels.datasets or your file)*

*# 2. Fit model, calculate T_n, detect change point.*

*# 3. Print and plot results.*

pass

def analyze_nikkei():

"""Apply SVR-SN to the Nikkei 225 data."""

*# [YOUR IMPLEMENTATION HERE]*

*# 1. Load your Nikkei return data.*

*# 2. Fit model, calculate T_n, detect change point(s).*

*# 3. Print and plot results.*

pass

*# -------------------------------*

*# MAIN EXECUTION BLOCK*

*# -------------------------------*

if __name__ == '__main__':

print("SVR-SN Algorithm Support Code")

*# Uncomment the sections you wish to run*

*# run_all_simulations() # WARNING: This is computationally intensive*

*# analyze_nile()*

*# analyze_nikkei()*

print("Code execution complete.")
